# Caring for people with disability and human growth: evolutionary perspectives and contribution to psychological wellbeing

**DOI:** 10.3389/fpsyg.2024.1371436

**Published:** 2024-06-24

**Authors:** Javier Bernacer

**Affiliations:** ^1^Mind-Brain Group, Institute for Culture and Society (ICS), University of Navarra, Pamplona, Spain; ^2^International Center of Neuroscience and Ethics (CINET), Tatiana Foundation, Madrid, Spain

**Keywords:** ableism, care, eugenics, flourishing, moral utilitarianism, natural selection

## Abstract

From an evolutionary point of view, organisms with mutations resulting in maladaptation are an unavoidable result of genetic variability, and they do not usually survive natural selection. Thus, they do not produce benefits for the species. I contend that this is different in humans at two levels. First, the existence of people with disability has been essential for human growth as a species. Human ancestors' evolving cognitive and social abilities were boosted by caring for vulnerable members of the species, including premature offspring and people with disability. Therefore, caregiving was an essential trait of the evolution of humans, intertwined with the development of bipedalism, the hand, face, vocal apparatus, and brain. Second, caring for disability is also a source of growth at a personal level. Even though most scientific literature focuses on the stress and burden caused by caring for people with disability, there is solid evidence to accept caregiving as a source of happiness and flourishing for human beings. Hence, disability still has an essential role in improving human life nowadays. Contrary to this evidence, influential utilitarian bioethicists promote the elimination of disability from modern societies. Following the arguments presented here, this will lead to the withering of society. In conclusion, disability should be acknowledged as an essential source of growth for the human species.

## 1 Introduction

According to the World Health Organization (WHO), about 16% of the world's population (1.3 billion people) experience significant disability (World Health Organization, 2022). The WHO and the United Nations, through the Convention on the Rights of Persons with Disabilities (www.ohchr.org), stress the importance of guaranteeing fundamental rights and equity for all human beings, irrespective of their physical or mental condition.

However, there appears to be a tension in the view on disability, spanning from a utilitarian to a humane stance. Some authors argue that dedicating vast resources to people whose quality of life would probably be low is morally wrong (McKie et al., 2016). On the other hand, other authors contend that we have the moral obligation, as human beings, to accept diversity and ensure the best possible life for any person (Bennett, 2014). Albercht and Devlieger coined the term “the disability paradox” to refer to the opposite opinions that people with disabilities and their families have about their lives in comparison with persons away from disability (Albrecht and Devlieger, 1999). According to Livneh (2012), the contributing factors to negative social attitudes toward disability are the following: (1) The standards of “beauty” institutionalized in society; (2) Emphasis on personal productiveness and achievement; (3) Socioeconomic factors (i.e., societal development, rate of unemployment, the importance of the nation's welfare economy); (4) The view on people with disability as a permanently sick person; (5) The stigmatization of a person with disability as an “outsider,” a deviant from the norm. Polarized positive and negative attitudes are frequent in different collectives, such as healthcare professionals (Friedman, 2023), children (MacMillan et al., 2014), high school and university students (de Laat et al., 2013), and older adults (Shimizu et al., 2023). Confirming the “disability paradox,” most reports show that respondents in contact with a person with disability have more positive attitudes (see Wang et al., 2021 for a recent systematic review), and an appropriate explanation of what disability is and how people with disability live are critical factors to change toward more positive attitudes (for example Daruwalla and Darcy, 2005; Krahé and Altwasser, 2006; Sullivan and Masters Glidden, 2014; Fisher and Purcal, 2017; Lautenbach and Heyder, 2019). Therefore, attempts to explain disability more accurately at all levels may help achieve more positive—and realistic—attitudes toward people with disability. Further, I will propose that these realistic and positive attitudes are intimately related to personal growth.

The main goal of this article is to show the essential positive role of disability in personal growth, both in human evolution and at present. To do so, instead of focusing on the person with disability, I will spotlight the caretaker of vulnerable species members. This goal unfolds in two specific objectives: first, to demonstrate that caring was an essential trait in human evolution, without which there would not be human beings whatsoever; second, to expose the positive psychological effects of caring for people with disabilities, summarized as an increased meaning and purpose of life. Note that by showing a “positive role” most of this article adopts a utilitarian approach, that is, it argues to what extent people with disability benefit the human species. In many respects, I consider utilitarianism as a limited approach to discussing human dignity, since any human life has an intrinsic value apart from its utility for the species. However, I take this attitude here because most arguments against the value of disability are grounded on utilitarian principles. Hence, I assume the playground and rules of utilitarianism to pinpoint disability's relevance.

After this introductory first section, I will explain in the second section how the initial proponents of “social Darwinism” proposed that sick and disabled persons weakened the species, and therefore took the first steps of the eugenics movement of the 19^th^ and 20^th^ centuries. However, the evolutionary history of humans is unique, and intelligence co-developed with genetic, physiological, ethical and political traits. In the third section, the configuration of the human body—hominization—will be explained from a systemic perspective, stressing that all specific human traits were developed interdependently. This process led to a species-threatening condition, the narrowing of the birth canal, which was overcome by giving birth to premature children who needed a long care period. Therefore, the fourth section shows that caretaking is an essential trait of our evolutionary history, such as bipedalism, the hand, face, vocal apparatus, and an extraordinary brain. This goes beyond the thoroughly discussed topic of altruism in evolution, which depicts an already-evolved, fully rational human being that “negotiates” with their conspecifics. Then, I present evidence that caring was generalized and extended to the long-term and specialized care of people with disabilities at least 2 million years ago. By doing so, crucial skills were developed to pinpoint the evolutionary success of human beings. This demonstrates the historical value of caring in personal growth through evolution. However, what about its current role? Section five exposes that the lives of caregivers of people with disability go beyond stress and the feeling of burden since they experience a generalized feeling of happiness, purpose in life, and resilience—in other words, they grow as human persons. Thus, disability keeps improving human life nowadays. Finally, section six reflects on current moral utilitarian trends and their impact on society, which suggest eradicating disability to increase happiness and reduce suffering. Given the arguments in sections four and five, these trends are revealed as ideological, unscientific and dangerous for human flourishing.

Before unfolding these theses, there is an important methodological consideration: drawing causal conclusions from evolutionary studies is a risky enterprise. As stated by Robert Trivers (quoted by De Waal, 2008, p. 280), in evolutionary studies “[y]ou begin with the effect of behavior on actors and recipients; you deal with the problem of internal motivation, which is a secondary problem, afterward… [I]f you start with motivation, you have given up the evolutionary analysis at the outset” (Trivers, 2002). Following this advice, I discuss the archaeological evidence of the effect of behavior (in this case, caregiving of disabled individuals) in section four, and section five suggests that a possible internal motivator of this behavior, as experienced nowadays, is the personal growth of the caregiver. Altogether, this points to the causal role of caregiving of vulnerable conspecifics in the improvement of the human species, at least hypothetically.

## 2 Disability from an evolutionary perspective

The natural selection theory proposes that organisms with an adaptive phenotype are more likely to survive and have offspring. This “adaptiveness” depends on environmental demands: if external conditions change, organisms with certain traits “are selected” because they are fit to the new circumstances. For natural selection to work, genetic variability due to changes in DNA (i.e., mutations, polymorphisms, and heritable epigenetic mechanisms) (Klironomos et al., 2013) is necessary. One side effect of genetic variability is the appearance of organisms with a disability, that is, with an impaired capability with respect to the average member of the species. In this section, the key issues of natural selection will be summarized, together with the attitude of this theory's initial proponents toward people with disability. The following sections will invite the reader to change the focus from the individual with a disability to the person who cares for them to understand correctly the evolutionary role of disability in humans. This brings an important consequence, namely to overcome a negative interpretation of disability on evolution based on false ideas, and put forth a positive one considering the peculiarities of the human species.

Charles Darwin (1809–1882) deserves gigantic credit for discovering a natural history compatible with a field of knowledge that did not exist, namely genetics. However, the seed of his theory was planted a long time ago. The idea of an evolving nature was already present in presocratic philosophers, whose main discussion revolved around the immutable and the temporary. Closer to Darwin's era, Lamarck introduced the concept of “evolution of nature” decades before the publication of *The Origin of the Species*; up to this moment, “evolution” was restricted to the development of a living being. In present times, how is evolution defined? According to Ernst Mayr, “Evolution is change in the properties of populations of organisms over time” (Mayr, 2001, p. 8). This definition entails a crucial nuance that contradicts other more popular versions of evolution, such as Richard Dawkins', for whom the basic unit of evolution is the gene (see, for example, Dawkins, 2006). Going back to Darwin, his most remarkable contribution is not evolution *per se* but the discovery of its conditions of possibility. Hence, he proposed that every current species result from an ancestor: those ancestors, in the past, bifurcated to originate new species. How and why are those new species coming about? Simply by environmental changes, the survival of the fittest, and their subsequent reproduction.

Albeit ignorant of the laws of genetics, Darwin suspected that individuals within a species might have different characteristics transferred to their offspring. We know this is due to mutations and polymorphisms, large or small changes in the genome, which are transmitted to the progeny. This is one of the pillars of the theory of natural selection: genetic variation. Another one, as explained by Ayala, is that individuals within a species have a variable rate of success in reproduction: “Only a fraction of produced organisms come to adult age and breed (…) The process leads to a gradual increase of useful variations and the termination of those less useful or harmful” (Ayala, 2006, p. 203). Therefore, “natural selection is simply the process of differential breeding of alternative genetic variants” (Ayala, 2006, p. 204). The production of such genetic variants is due to seven possible processes (Mayr, 2001, p. 97–101): mutations (genomic changes), gene flow (between different populations of the same species), genetic drift (loss of alleles due to stochastic events), biased variation (distribution of alleles in gametes in more than half of the instances), transposable elements (DNA fragments that tend to move within the genome), non-random mating (preference for a mate with a particular phenotype), and the most relevant, natural selection. This has a negative interpretation since it refers to eliminating unfit individuals according to environmental changes. Initially, Darwin referred to this as a “struggle for existence” (Darwin, 1859, p. 60), although Herbert Spencer's expression became more popular: “survival of the fittest” (Spencer, 1910, p. 264).

The general picture, as viewed nowadays, is straightforward: within a species, individuals have a genetic variability that, together with changes in environmental conditions and differential reproduction, leads to a natural selection process that, as a consequence, eliminates unfit individuals. Only surviving individuals breed and transfer their genotypic and phenotypic traits to their offspring. Thus, from an evolutionary perspective, carriers of a severe disability are unfit to survive and to transfer their genetic traits. From the point of view of the species, their contribution to survival or adaptation is null. This is not an interpretation but a logical consequence of the theory of natural selection. In Sections 3 and 4, I will show that the emergence of human beings in natural history changed the rules of evolution in this respect. Before that, let us go deeper into the evolutionary understanding of disability.

At this point, we should distinguish the theory of natural selection from Darwin's personal opinion on disability, which is subsumed in a specific period of history when diversity was poorly accepted. On the other hand, his colleagues and disciples did not make this distinction and developed “social Darwinism,” which is one of the pillars of eugenics. Darwin clearly explained his vision of human disability in *The Descent of Man*, published in 1871. In Chapter V, about intellectual faculties, referring to “Natural selection as affecting civilized nations,” Darwin explains the following:

With savages, the weak in body or mind are soon eliminated; and those that survive commonly exhibit a vigorous state of health. We civilized men, on the other hand, do our utmost to check the process of elimination; we build asylums for the imbecile, the maimed, and the sick; we institute poor-laws; and our medical men exert their utmost skill to save the life of every one to the last moment. There is reason to believe that vaccination has preserved thousands, who from a weak constitution would formerly have succumbed to small-pox. Thus the weak members of civilized societies propagate their kind. No one who has attended to the breeding of domestic animals will doubt that this must be highly injurious to the race of man. It is surprising how soon a want of care, or care wrongly directed, leads to the degeneration of a domestic race; but excepting in the case of man himself, hardly any one is so ignorant as to allow his worst animals to breed (Darwin, 1871, p. 168).

According to Darwin, medical progress does not make sense in the light of evolution. In a systematic study of Darwin's interpretation of intellectual disability, Steven Gelb classifies it in four different ways: (1) as the intermediate step between human beings and other primates; (2) as an example of defective products of variability; (3) as the bottom step of all beings ordered by their intelligence; and (4) as atavistic reversions of the human species going back to extinct forms (Gelb, 2008). These ideas were expanded by Francis Galton and Herbert Spencer, who were very influential in the eugenic movement at the beginning of the 20^th^ century. Quoting Farrall (1985, p. 55), “Eugenics was in reality applied biology based on the central biological theory of the day, namely the Darwinian theory of evolution” (as cited in Davis, 2017, p. 4). Other scholars contend that “social Darwinism” is a perversion and misapplication of the natural selection theory (Dennett, 1995). This is reasonable, but it is undeniable that Darwin's inner circle developed this point of view about human society. As finely explained by Paul (2003, p. 235), “Darwin was thus not a ‘eugenicist,' or certainly not a fully-fledged one. But his theory fueled fears that made the need for a programme of selective breeding seem dire. It is no coincidence that Galton, the founder of modern eugenics, was his cousin—or that Leonard Darwin, President of the eugenics Society in Britain in the 1910s and 1920s, was his son.” This may seem an extreme perspective from a distant past when cultures were not ready for the diversity we experience today. However, it is far from an anachronism, as will be discussed in Section 6 of this manuscript.

This reading of Darwin's work and the inventors of “social Darwinism” (Galton, Spencer, Huxley, Malthus) is not new. Many scholars have related this kind of “social human natural selection” to ableism (see Branch et al., 2022 for a recent overview). This term was coined in the early nineteen eighties and points to the “discrimination against people who are not able-bodied, or an assumption that it is necessary to cater only for able-bodied people” (Honderich, 2005). This term is also commonly used to refer to intellectual disability. Nevertheless, other authors explain that this is a distorted interpretation of Darwin's writings (see, for example, Lau, 2022), although the role of “social Darwinism” in eugenics is undisputed. In Lau's opinion, the British biologist did not believe that individuals of a species had an ideal configuration. Therefore, any “impairment” just showed variability of a particular trait, not a disability. Also, Lau reinforces this idea by explaining Darwin's need to be supported by some of his contemporaries since he considered himself unable—disabled—to develop his theory. Be as it may this section briefly shows that, according to the theory of natural selection, maladapted individuals resulting from genetic variability do not contribute to the success of the species. In Sections 3 and 4, I will turn to the scientific facts of human evolution, specifically regarding the conformation of the body and the survival of our ancestors. This intends to show that, irrespective of the role of disability in evolution in general terms, it plays an essential role in the case of humans due to a radical trait of our evolution: caring for the vulnerable.

## 3 Human evolution: the need for a systemic approach

We must assume a systemic approach to understand the importance of caretaking on current human beings and our ancestors. This entails the fact that the distinctive features of the human body did not appear individually but as a complex system. Thus, all of them are interdependent, and the lack of one of them would change the actual configuration of the body completely. In this context, “essential trait” refers to any of these individual features that cannot be split from the whole system (i.e., the human being) without essentially affecting it. Some of these features are anatomical (such as bipedalism, the hands or the development of the vocal apparatus), while others are cognitive (intelligence) or relational (caregiving). Remarkably, the systemic approach seeks to soften the sharp distinction of these categories (anatomical, cognitive, and relational) precisely because of the inherent interdependence of the traits.

The final bifurcation that set the human and chimpanzee lineages apart happened between 5 and 8 million years ago. Australopithecines appeared first, and two million years ago, the genus *Homo* followed their lead. Which of these antecessors can be considered human is a debated issue and unimportant for the sake of this manuscript. In any case, it is worth noting that 2 million years is a mere instant in the context of the evolution of life. As far as we know, humanity did not experience essential changes during that period. However, the invention of agriculture (about 12,000 years ago) led to the cultural revolution that allowed the adaptation of the environment to human life, not vice versa. Most experts agree that human cultural evolution is a hotspot in the evolution of life (see part III of Avise and Ayala, 2010). As Graeber and Wengrow (2021) put it, in the Spanish caves of Altamira, there are paintings from 25,000 b. C. to 15,000 b. C. Many things could have happened during those ten thousand years, but it is hard to believe that the world changed as it has since 8,000 b. C. to today.

How were the gradual set of changes that transformed our common ancestor with chimpanzees to the human lineage? Lombo and Gimenez-Amaya (2016) propose that the primary organic traits that distinguish humans from other animals are bipedalism, the hands, the face, the vocal apparatus, and the brain. The key idea, once again, is that all these traits evolved systemically through millions of years; otherwise, they would not be part of our bodies nowadays. Humans are the only animals considered fully bipedal (Alexander, 2004). Even though birds, kangaroos, and some apes can walk with only their lower limbs, true bipedalism entails skeletal and muscular changes that impact the whole body and make humans unfold their daily lives in an erect posture. Different hypotheses try to explain how our ancestors became bipedal or, in other words, how locomotion was in our common ancestors with other apes. Whereas some initially relied on environmental changes transforming forests into open savannahs, this hypothesis was discarded due to newly discovered fossils (Cela-Conde, 1996). Currently, the two competing models propose that bipedalism occurred either as an adaptation of arboreal movement (Stamos and Alemseged, 2023) or in an already terrestrial animal that habitually knuckle-walked (Richmond et al., 2001). In any case, the most apparent physiological trait that accompanied bipedalism was the co-evolution of the hands, as Darwin already noted in systemic terms: “As the progenitors of man became more and more erect, with their hands and arms more and more modified…” (Darwin, 1871, p. 143). The hands have been a longstanding key issue in the development of human rationality, as presocratic philosophers already viewed it: “Now it is the opinion of Anaxagoras that the possession of these hands is the cause of man being of all animals the most intelligent. However, it is more rational to suppose that his endowment with hands is the consequence rather than the cause of his superior intelligence” (Aristotle, 1961, book IV 10, 687a). The uniqueness of the human hand has been summarized in three abilities that are absent in other primates (Kivell, 2015): (1) precision handling, (2) forceful precision gripping, and (3) power squeeze gripping of cylindrical objects. This allowed our ancestors to throw stones and wield clubs (Young, 2003) and develop creativity through tool-making (Davidson and McGrew, 2005).

Bipedalism entails a unique configuration of the whole body, from feet to skull. The weight of the entire body must be supported by the inferior limbs, resulting in a “larger femoral head, increased femoral neck length, anteroposteriorly elongated condyles of the femur, the knee positioned in slight valgus at knees due to bicondylar angle, shorter big toe and a higher foot arch.” (Yavuzer, 2020, p. 489). Also, the foramen magnum appears in a central position, which allows the skull (and brain) to grow in all directions. Before focusing on the brain, there is another organic trait usually overseen in this context, although crucial to understand the evolution of human beings: the face. Recent research explains in detail how the face evolved among our ancestors (Lacruz et al., 2019), which is beyond the scope of this article. For the sake of our argument, the key idea is that the face evolved systemically with the other organic traits, which was crucial in social communication, as highlighted by Lacruz and collaborators. The progressive softening of the “snout” and its transformation into an expressive face was possibly due, among other factors, to the progressive use of the hands to separate the edible from the non-eatable hard parts of food, as well as to bring water to the mouth with the same method. Hence, this human ancestor was standing up on their lower limbs, using the hands for increasingly complex tasks, with the concomitant development of intelligence, and being able to communicate emotions through a more and more flexible face. Furthermore, organs in the anterior part of the body gradually re-organized, allowing the development of the vocal apparatus. The human uniqueness of this anatomical trait is also unquestioned and summarized in three issues: the descended larynx, increased thoracic innervation and breath control, and laryngeal air sacs (Ghazanfar and Rendall, 2008). This allows the production of complex sounds that, ultimately, become language.

Bipedalism, hands, face, and vocal apparatus are unique anatomical and physiological traits that coevolved as a whole in our ancestors. This evolution was also accompanied systemically by psychological capacities, such as communication, emotional awareness, and, in general terms, intelligence. In close relation to this, the brain also experienced a unique configuration of its anatomy and, presumably, function. The first evident change in the human brain through evolution is its increase in size: the human brain is the largest in the Hominidae family, and cranial records demonstrate an increased size from australopithecines to *Homo sapiens* (Smith and Tompkins, 1995). Moreover, the distinctive feature of the human brain concerning this is not the absolute size but the highest encephalization quotient (i.e., brain/body relationship): in hominids, there is a disproportionate enlargement of the brain with respect to a relatively small increase in body size, and this is not the case in australopithecines and extant great apes (Roth and Dicke, 2005). Apart from this noticeable difference, several brain regions are also expanded in humans in comparison with our ancestors and other members of our evolutionary family (see Schoenemann, 2006 for a review): different areas of the frontal cortex (Preuss and Wise, 2022) [for instance, the frontal pole (Semendeferi et al., 2001) or Broca's area (Rilling, 2014)], parietal lobe (Bruner, 2018), cerebellum (MacLeod et al., 2003), hippocampal complex (Schilder et al., 2020), or amygdaloid complex (Barger et al., 2007). On the other hand, other areas, such as the olfactory bulb or visual cortex, have a reduced size or are smaller than expected (Schoenemann, 2006).

Many other changes in the human nervous system can be ascribed to evolution, for instance, the proliferation of dendritic spines (DeFelipe, 2011) or differential gene expression (Sousa et al., 2017). A systematic review of these changes is unintended for the present contribution. Going back to the critical point of this section, all these neural traits should be viewed as part of the systemic evolution of the human being: the body evolves as a whole. Further, this “physiological” evolution is also inextricably accompanied by a psychological evolution. It is hard to believe that the evolution of intelligence was independent of anatomical changes. Recent research confirms Anaxagoras' hypothesis about the interdependence of hand use and intelligence (Kulik et al., 2023), and the “social brain hypothesis” (primates have larger brains than expected to manage complex social relationships) is widely accepted (Dunbar, 2016). Following this co-evolution of physiological and psychological traits, the next section will explore the role of care in the evolution of the human being. The hypothesis is that caring for others is another systemic (and therefore essential) trait of human evolution: if evolution were rewound and repeated without it, *ceteris paribus*, the final result would be a completely different “human” being. As a matter of fact, there would probably not be humans on Earth.

## 4 The essential role of care in evolutionary human growth

The systemic development of the human species involved an outstanding challenge, nearly impossible to assume from an evolutionary perspective: bipedalism resulted in a narrowing of the birth canal, so the survival of female individuals and the offspring hung by a thread. How could pass the test of natural selection a trait that threatened the survival of the whole species? This is known as the “obstetric dilemma” in human evolution.

However, the benefits of bipedalism had to be so high that an alternative pathway was naturally selected: the offspring was given birth prematurely to increase the probability of survival, both of the newborns and their mothers. Other authors link human prematurity to the mother's metabolic demands (Dunsworth et al., 2012), that is, the capacity of the mother to support her metabolic demands and those of the fetus. For the sake of my arguments, both hypotheses are similar. Adopting a systemic stance, both can be viewed as different narratives of the same fact: to balance some evolutionary demand (i.e., bipedalism or metabolism), human childbirth happens prematurely.

The human ancestor co-developing some unique traits such as an enlarged brain, an expressive face, a progressively complex vocal apparatus, and outstanding intelligence compared to other animals, also had to extend caretaking of the offspring. Due to our mammal condition, breastfeeding favored the mother as the primary caretaker. Consequently, the mother had higher postpartum metabolic demands, and her abilities to fulfill them were compromised. Thus, the group established a supportive structure for the mother to secure the baby's development (Nowell and Kurki, 2020). The human species has a high degree of “alloparenting” (i.e., children are cared for by other group members apart from the parents, such as brothers and sisters, grandparents, etc.) even in its first evolutionary steps (Kenkel et al., 2017). In conclusion, caretaking of the temporally vulnerable members of the species (i.e., the baby and puerperal mother) is another essential trait in systemic human evolution, connatural to bipedalism, an expressive face, a complex vocal apparatus, and a unique hand, brain, and intelligence.

Let us move one step forward. The temporary caretaking of the offspring or their mothers may be acceptable, but what about caring for disabled individuals, which may require constant, lifelong, and possibly specialized attention? The generalized viewpoint on our ancestors is that they were brute, wild, and violent people only interested in their survival. Graeber and Wengrow (2021) offer a “new history of humanity,” dismounting these clichés inspired by Rousseau's and Hobbes' philosophy to justify the worst version of colonialism. They describe the history of several human groups worldwide as told by indigenous thinkers, avoiding Occidentalism and a politically-biased interpretation. Part of this “new history of humanity” involves describing archeological sites where the caretaking of people with a disability is proven. For example, they mention an Italian archaeological dig, Romito 2, where the burial of a person with acromesomelic dysplasia—a sort of “dwarfism”—was found. This human group is estimated to have lived in the area about 10,000 years ago, and the buried person died at 17–20 years old. This means that the group took care of her/him for all those years, providing food and support until she/he reached adulthood—life expectancy during the Neolithic era was 20–33 years.

The precise way in which caregiving of the offspring and puerperal mothers was generalized to people with disability is unknown, but the archaeological evidences show that it actually happened: The case of Romito 2 is far from being an exception. Lorna Tilley (2015; see Table 2.1 in her book) details 38 similar cases, spanning from 1,77 million years ago to the 17^th^ century of our era and covering human groups around the globe. In her words, these examples “represent a very small proportion of the thousands [of reports] that document human remains displaying evidence for a period of survival with severe and likely disabling pathology.” (Tilley, 2015, p. 29). She complains that the archaeology of care draws limited attention, despite the solid evidence to support it. According to Tilley, in any case, whether caretaking should be considered a biological evolutionary trait is unclear: “because even where the detail necessary to make an assessment of such a claim is provided, there are no behaviors ascribed to biology that are not equally well or better explained by sociocultural learning processes” (Tilley, 2015, p. 102). In my opinion, a dichotomist choice between “nature and nurture” is unnecessary on this occasion since both options are acceptable: caretaking is essential during hominization because it guaranteed survival of the offspring and their mothers despite the “obstetrical dilemma;” further, habits and attitudes unfolded after this family caretaking were generalized toward other vulnerable members of the group, either children or adults, which enhanced the qualities that made us the most successful species on Earth: an emotion-imbued intellectual knowledge. In the same line, Winder and Winder (2015) propose that caring for other vulnerable members of the group was the key evolutionary trait of humans. According to them, the life of ancient humans in small groups should entail a high disability rate due to consanguinity. In this situation, evolutionary pressure involved the best strategies to deal with disabilities, both one's own and those of other group members, developing enhanced intelligence, cognitive flexibility, and compassion. Hence, Winder and Winder pose the “vulnerable ape” hypothesis to explain the first steps of our ancestors toward humanization. Kessler et al. (2018) agree on relating group size and caregiving as critical issues for the evolution of the first species of the genus *Homo*.

Some of the topics presented in this section have been extensively discussed under the umbrella of “altruism” in evolution. Further descriptions of the topic can be found elsewhere (for example Fehr and Fischbacher, 2003; De Waal, 2008; Warneken and Tomasello, 2009; Vlerick, 2021). In my opinion, the presentation of caregiving as an essential trait inseparable from the evolution of the human body—and, therefore, from the origin of the human person—goes beyond the definition of altruism as the “instinctive cooperative behavior that is detrimental or without reproductive benefit to the individual but that contributes to the survival of the group to which the individual belongs” (Tilley, 2015, p. 102–103), “a behavior that benefits others at the cost of the lifetime production of offspring by the altruist” (Tilley, 2015, p. 103), or “behavior that increases the recipient's fitness at a cost to the performers” (De Waal, 2008, p. 280). However, it gets closer to this quotation of Mayr's work: “The traditional definition of altruism is that it consists of an act that is beneficial to the recipient but is performed by the altruist at a cost. This definition excludes all kindness and helpfulness that is performed without noteworthy cost. Yet, in a social group much behavior consists of acts of kindness and thoughtfulness that are performed without any noticeable costs. And it is precisely this kind of behavior that is not only very important for the cohesion of a social group but that also forms a bridge to strictly defined altruism” (Mayr, 2001, p. 257). Whereas most descriptions of altruism in evolution depict a cold-blooded human agent that weighs the costs and benefits of cooperation, it is more realistic to understand caregiving of in-group vulnerable members as a natural behavior where costs and benefits are not weighed or even considered, but just part of life. Thus, even though caregiving and altruism may be related topics, it is convenient to demarcate their differences to understand them in depth. Going back to Mayr (2001, p. 132): “The altruism that members of a social group show to other related members of the group (excluding offspring) is apparently never anywhere near as great as the altruism displayed by parents (particularly mothers) to their own offspring.”

To have a holistic view of the implications of caretaking for humans, let us do a brief theoretical analysis of care. The study of care as a topic of philosophical reflection is relatively recent, dating back to the eighties of the 20^th^ century. Considering what has been explained above, it is unsurprising that this topic emerged from the feminist perspective of authors such as Carol Gilligan and Virginia Held. The dominant theories of moral development during that time, with postmodernism at its peak, praised the Enlightenment values of autonomy, freedom, and independence. Gilligan condemns that these theories do not represent the experience of human beings, especially women, for whom interpersonal relationships, empathy, and mutual care are much more relevant (Gilligan, 1982). Held develops these ideas by setting the mother-child relationship as the paradigmatic case of how mutual relationships are enrooted in human development (Held, 1987) and criticizes that societies are built upon “contracts,” as proposed by Hobbes and Rousseau. According to Gonzalez and Iffland, these perspectives invite to approach anthropology in a very different way: “Ethical theories that privilege abstract notions of ‘justice' or ‘right,' principles of harm and beneficence, or general moral rules fail to account for certain facts about the lived experiences of human beings or, at least, fail to account for the kinds of lives experienced by women—lives (of both men and women, young and old) affected by extended periods of bodily infirmity, dependence, and vulnerability. If we are attentive to these latter kinds of relationships, how do our ethics change?” (Gonzalez and Iffland, 2014, p. 11). These authors stress that, whereas “contractual” relationships à la Rousseau or Hobbes involve autonomous relationships between equals, maternity points to utterly unequal and usually involuntary relationships since no one chooses to depend on anyone: it just happens, and there is no weighing of costs and benefits. When we become aware of all this, i.e., that society is not built on the foundations of freedom and independence but on those of vulnerability and dependence, a new set of values arises, constituting the ethics of care. This does not mean that everybody needs to care for someone to be human, but it stresses that caring for someone is natural and good.

However, is it true that caring for someone is *good*? Could this be an empty philosophical reflection alien to empirical evidence? The fact is that, when reviewing the scientific literature on the effects of taking care of people with disability, results point to two words: stress and burden. How could a stressful burden lead to human growth?

## 5 The psychological life of caregivers: a unique opportunity for growth

The scientific method requires establishing a hypothesis to be confirmed or falsified after collecting and analyzing the evidence. Hence, results depend on the hypothesis to be tested. Suppose the hypothesis is that parents and caregivers of children with disability experience stress and a feeling of burden, and they are only asked about their stress and feelings of burden. In that case, the hypothesis will unquestionably be confirmed. As illustrated in Figure 1 by Marino et al. (2017) (based on Google Books Ngram Viewer, which shows the percentage of books published on the topics selected per year), most scientific literature on caregiving has concentrated on burden and stress. Updating their data, in 2019, about 72% of publications on these topics were about burden, 25% about stress, and 3% about the positive effects of caregiving. On the other hand, when some questions about, for instance, coping strategies to overcome the feelings of stress and burden are included, the researcher starts finding a different picture. For example, Sandilands et al. (2022) conducted a literature review on the burden in primary caregivers of children with inherited rare diseases. They built a conceptual model to systematize the source of burden in these cases. Further, they highlighted the coping strategies that caregivers develop; in other words, the human growth that they experience as a consequence of caring for a child with a rare disease: acceptance, support systems (i.e., interpersonal support caregivers receive from different groups, such as co-workers, friends, family, health professionals, etc.), gratitude and hope, faith, quest for knowledge, and successful routine establishment.

Other authors have already exposed the need for a change of perspective. Marino and collaborators (2017) carried out a thorough literature review on hedonic and eudaimonic experiences by caregivers of family members. For clarification: “hedonic” refers to pleasure, enjoyment, or comfort, and therefore is considered to be short-termed; “eudaimonic” is related to self-satisfaction with life through skill and virtue development, personal growth, flourishing, etc. (about the relationship between eudaimonia and personal growth, see Ryff, 2014). Interestingly, their review shows that the negative effect on hedonic markers does exist in caregivers, albeit with a tiny statistical effect size primarily led by study design. Moreover, depressive symptoms may be related to having a relative with a severe disease rather than caring for them (see also Amirkhanyan and Wolf, 2003). Conversely, eudaimonic (long-term) assessments are enhanced in caregivers, who experience higher purpose in life, personal growth, environmental mastery, positive relationships, and self-acceptance.

This effect is more pronounced when caring for children with disability. Young et al. (2020) explored the vital experience for 1 year of 28 mothers and five fathers raising a child with disability. Grief and anxiety were present but progressively decreased and became intermittent. Personal growth was also typical, defined as developing strategies to cope with negative feelings. Findler (2014) compared the stress and personal growth of grandparents of children with (*N* = 94) and without (*N* = 105) intellectual disabilities. Interestingly, negative feelings were significantly increased in grandparents of children without disability. As the author states, “This study aimed to correct the nearly exclusive focus in the literature on negativity, stress, and cost of grandparenting children with disabilities, as well as to test the pervasive assumption that the absence of disability results in an almost entirely positive grandparenting experience with nearly no negative affect.” (p. 32).

As exposed above, most research articles are hypothesis-driven toward the adverse effects of caring for disability. Thus, it is legitimate to turn to publications testing the hypothesis that caring for disability is positive. Remarkably, Stainton and Besser (1998), after interviewing 17 families and applying qualitative analysis, point to eight life domains where parents of children with intellectual disability feel privileged. First, they perceive their children as a source of happiness, mainly because they see them doing things considered impossible initially. Second, they point to an increased sense of purpose and priorities. As one interviewee states, “It simplifies life. The basics which is, you know, love and affection and being there. You know what I think, he's made our lives better in a way. It makes you realize what's important in life, you know, it's not how many things you have, or where you live… what's important is caring for people or being sensitive to others” (Stainton and Besser, 1998, p. 62). Third, they also emphasize the expanded personal and social networks: “…we've become the people we always hoped to be… involved in your community” (p. 63). The fourth area refers to increased spirituality, which is the most heterogeneous field: the critical point appears to be a *change* in spirituality, so the result depends on the family's starting point. This is of great interest because it shows that attitudes toward disability are not necessarily driven by religion or spirituality, as commonly believed. Fifth, the child is viewed as a source of family unity: “He has brought us closer and both our daughters are taking part in the process” (p. 64). In any case, interviewees recognize that it is a challenging “make or break” experience. The sixth theme refers to children being the source of increased tolerance and understanding, not only about disability but also about diversity in general. This is especially present in siblings. Seventh, they are also perceived as a source of personal growth and strength, stressing perseverance and the feeling of being more prepared than other families for unexpected challenges: “It gave us more strength, and we'd go and say, ‘way to go, Caroline, you know. Look at what you have made of us. You made us door openers.' And we're getting really good at it” (p. 65). Finally, they point to the positive impacts on others and the community. As happened in the first theme, community members also experience a rewarding effect when seeing children with disability get unexpected achievements. As an example, a family tells how no one wanted to teach the Bible to their son, so he learned it by himself for his Bar-Mitzvah: “I looked at him standing at the Torah and said: ‘he is doing what every other 13 year old Jewish boy does, exactly'… People we didn't know were crying because it was an amazing achievement” (p. 66). It is worth noting that the only theme where families have a negative experience is the interaction with professionals and services. The common issue in this topic is the feeling of being victimized and the brutality when communicating diagnosis: “The doctor told me: ‘wipe the smile off your face, I'm gonna tell you something.' I thought oh my God, the poor kid, he said ‘never mind the poor kid, poor you.' Imagine, and this was one of the top pediatricians in the territory” (p. 67).

The positive effect of caretaking vulnerable people extends to other realms, such as healthcare professionals giving palliative care to children (Beaune et al., 2018). These authors acknowledge that the positive psychological effects of medical caregiving are still unexplored. In their study, all professionals (*N* = 25: 9 social workers, eight nurses, eight physicians) consistently report that their work has promoted a change of perspective in their lives, led by higher gratitude, acknowledgment of strength and resilience in others, and redefinition of priorities. This new perspective is summarized with the following sentence: “I enjoy watching families at their worst and watching them change and develop and find skills within them[selves] that they never thought they had.” (Beaune et al., 2018, p. 5). Also, they report an enhancement of their resources far beyond what they learned at the university, including aspects on the end of life and a “good death:” “What is a good death? You know, surrounded by a loving family, pain-free, full explanations, parents fully understanding, with whatever religious ceremonies they want. That's what we need to work toward.” (p. 6). The last topic is benevolence, understood as contributing to something good and valuable through your work. Overall, it is described as the feeling of having given everything they could for those children and their families. These attitudes are similar to those reported by Satchidanand et al. (2012) by students of healthcare professions in contact with patients with physical disabilities. Results point to a positive attitude, enhanced when students have had more professional or personal experience with people with disability.

Unquestionably, caring for vulnerable people is a source of stress and the feeling of carrying a burden that, at some point, seems unbearable. Health systems and public and private associations should increase resources to minimize these adverse effects. On the other hand, scientific evidence strongly suggests the positive impact of these situations on the personal growth of professionals and families. In summary, this personal growth is synthesized as the increased realization of priorities in life, focused on loving others; enhanced resilience and perseverance in the face of difficulties; increased gratitude and hope; better understanding of diversity and vulnerability; stronger social bonds; and, overall, the feeling of making sense of life. Considering the development of these virtues, it is unsurprising that philosopher Margaret Archer stated that caregivers of vulnerable people “may be the only experts on being human.” (quoted by Gonzalez and Iffland, 2014, p. 21).

## 6 Current directions: toward a withered society?

This last section shows a brief diagnosis of how people with disability are viewed according to some bioethical trends. In section number 2, I mentioned how the eugenics movement by the end of the 19^th^ and first half of the 20^th^ century arose, at least in part, from social Darwinism. This looks brutal and unacceptable in the era of political correctness. Or is it?

Utilitarian moral philosophy is widely accepted in bioethics (see for example Savulescu and Birks, 2012). According to it, promoting wellbeing—usually understood from a hedonic point of view—and reducing suffering for as many people as possible is morally good. Considering this, the extreme branch of utilitarian bioethics proposes the elimination of people with disability since it is identified with pointless suffering. The link between this ethical standpoint and evolution is common, as done by John Harris in his book *Enhancing Evolution*: “I have attempted to explain how abortion and even infanticide for disability are extensions of the legitimate, perhaps imperative, ethic of combating disability” (Harris, 2007, p. 100). According to him, this is the means to boost human evolution, which we are morally forced to pursue. This is a standard view in scholarly circles but also disseminated to society by champions of scientific communication, such as Richard Dawkins or Peter Singer. The former is well known for his strong eugenic opinions, such as the controversy created on Twitter when he stated that it would be immoral not to abort a child with Down syndrome (see, for example, The Guardian, 2014 for a complete description of the controversy). As he explained in more detail, his rationale is that Down syndrome only brings suffering and not happiness to the patient and their families, and therefore it is immoral to let these children exist. Although governments obscurely report the official figures of abortion rates by condition, Dawkins' ideas appear to be widely accepted by society nowadays: reduction of live-birth prevalence estimates due to Down-syndrome-specific elective abortion is, on average, 54% in Europe (de Graaf et al., 2021), reaching 87% in Spain. In the USA, figures range from 67% to 85% (Natoli et al., 2012). Further, abortion is progressively more frequent in mild malformations such as cleft lip or palate.

Peter Singer is another popular representative of utilitarian moral philosophy. He goes one step further and defends that society should also be able to eliminate children with disability after birth, which is usually referred to with the euphemism “postnatal abortion.” In his famous work *Practical Ethics*, he proposes that “defective infants lack these characteristics [rationality, autonomy, self-consciousness]… Killing them, therefore, cannot be equated with killing normal human beings or any other self-conscious beings” (Singer, 1979, p. 121). Considering the findings of the previous section of this article, this utilitarian view of disability is ideologically biased and alien to scientific evidence (see also Vehmas, 1999): caring for the vulnerable is a source of happiness beyond any other experience. Maybe the followers of utilitarian bioethics are considering the economic impact of caring for someone who does not “produce profits” also in economic terms to society. Given that caregiving promotes human growth, they should consider the long-term benefits of the process. In this manuscript, I have focused on the impact of disability on the caregiver. Whether the person with disability suffers more than other people or more than precluding them from being born is hard to assess. However, it is evident that suffering is intrinsic to human life and may be alleviated by support, respect, and love.

Some readers may think that “postnatal abortion” is a theoretical proposal by a philosopher that could never be realized in a humane world. This is not the case. The Groningen Protocol allows “euthanasia” for children under 1 year in the Netherlands (Verhagen and Sauer, 2005). It is confusing to use the term “euthanasia” in this case because euthanasia usually involves a decision by the person to face death. The Protocol can be applied if the following conditions are fulfilled (Kon et al., 2022): (1) The diagnosis and prognosis must be certain; (2) Hopeless and unbearable suffering must be present; (3) The diagnosis, prognosis, and unbearable suffering must be confirmed by at least one independent doctor; (4) Both parents must give informed consent; and (5) The procedure must be performed in accordance with the accepted medical standard. Given the two first points, the Protocol is impossible to be applied. On the one hand, medical diagnosis cannot be certain (Balogh et al., 2015): “Absolute certainty in diagnosis is unattainable, no matter how much information we gather, how many observations we make, or how many tests we perform” (Kassirer, 1989; as cited in Balogh et al., 2015). On the other hand, Kon and colleagues define “unbearable suffering” as “subjective suffering to the extent that the patient herself feels that she can no longer bear it, and she believes that being dead would be better than being alive in her current state. That is, a degree of suffering that to the patient constitutes a fate worse than death” (Kon et al., 2022, p. 292, footnote 2). It is unreasonable to think that a baby under 12 months could report “unbearable suffering” as defined here, so condition #2 is also impossible to fulfill. Remarkably, selective abortion of children with potential disabilities and the Protocol of Groningen violate Article 10 of the UN's Convention on the Rights of Persons with Disabilities (Right to Life), which reads: “States Parties reaffirm that every human being has the inherent right to life and shall take all necessary measures to ensure its effective enjoyment by persons with disabilities on an equal basis with others.”

Condition #3 is also disputed since physicians could have a biased opinion on the patient with disabilities. Some studies show that over 80% of physicians treating people with disabilities consider the patient to have a poor quality of life (Gill, 2000). As exposed by Stainton and Besser (1998), interviewees unanimously reported that the only negative issue of having a child with disability was dealing with the negative opinion of healthcare professionals. As mentioned in the first section of this manuscript, the “disability paradox” shows the opposite opinions of people with disabilities and their families compared with those who never experienced it (Albrecht and Devlieger, 1999). Whereas the former consider the possibility of searching for happiness as any other human being through identity empowering and social relationships, the latter believe that a life with disability is not worth it. The core of the problem is that laws, regulations, and decision making at the macro level are usually dictated by people far away from disability, so it is profoundly biased toward one side of the paradox. Stainton recently expressed the concern of several associations of people with disability toward the new regulations regarding euthanasia and assisted suicide (Stainton, 2022). He says the main issue is the false negative view of disability, leading society to “modern quiet” eugenics. This has been denounced in the past, such as the anti-eugenics movement by people with cerebral palsies in Japan. According to Tagaki (2023), this group reinforced their identity by distancing from the common view that only able-bodied individuals can be happy. In any case, from a medical point of view, disability should be viewed as a pathological condition to be treated and, if possible without affecting human beings, cured. A utilitarian logical (but absurd) consequence of my arguments is that disability should be enhanced in humans since it improves the human species. This is another proof that utilitarian bioethics are limited to understanding certain human aspects. All efforts should be made to cure, alleviate and improve the quality of life of people with disability and their caregivers. In the meantime, research should expose their actual experience from all corners.

As a final idea, the disability paradox may be due to a radically different interpretation of disability by people with and without it. Douglas Baynton has conducted an extensive sociological study of the term (Baynton, 2001). He says the tag “disability” has been used to justify social inequality in the USA. He focused on three historically mistreated groups in this country: women, the black community, and immigrants. In his opinion, the standard strategy to deny fundamental rights to these groups has been to consider them as “disabled.” In the case of women, opponents to their equality with men argued that they were physically frail, irrational, and emotionally unstable, and therefore they could not vote adequately. Slavery, due to racial reasons, was dogmatically justified because black people were considered as poorly evolved human beings, intellectually handicapped, and predisposed to mental and physical disorders, as well as immoral behaviors. Concerning immigrants, regulations established quotas for several ethnic groups or nationalities and stated that some “defective races” were prone to congenital defects. Baynton's primary interest is not to denounce these atrocities, which are quickly deprecated, but to show how *disability is a legitimate reason for inequality*. Nowadays, we are scandalized that these arguments were used to marginalize women, non-Caucasian people, or immigrants. Maybe we should also be outraged for using it to deny fundamental rights to discriminate against people with diverse conditions. Firm steps are being taken to enhance the inclusion of people with disabilities in fields such as creativity, for example (Jones, 2022). This shows that the ethical attitude toward some groups depends on the common goods sought by society: if the community is only moved by survival, reproduction or hedonism, the most vulnerable groups will be marginalized. However, they will be affectionately cared for if humane values are defended, leading to a societal flourishing.

Considering the outstanding human growth that produces caretaking of vulnerable people at evolutionary, social, and personal levels, it is inevitable to put the tag of “withered” on a society that intends to erase vulnerable people. If functional diversity were eradicated and the “ideal society” proposed by utilitarian moral philosophers and transhumanists were achieved, those “human beings” would lack compassion, purpose in life, and resilience. Instead of growing, they would wither. Suffering would eventually happen, and they would be poorly prepared to face it.

It would be hard to consider such a society as human.

## Data availability statement

The original contributions presented in the study are included in the article/supplementary material, further inquiries can be directed to the corresponding author.

## Author contributions

JB: Conceptualization, Investigation, Writing – original draft, Writing – review & editing.
